# Life-Course Framework for Institute-Based Surveillance of Overweight, Obesity, and Metabolic Risk Factors in India: Protocol for Surveillance System Development

**DOI:** 10.2196/86084

**Published:** 2026-04-13

**Authors:** Sai Ram Challa, Biswanath Dash

**Affiliations:** 1 Department of Humanities and Social Sciences, Birla Institute of Technology and Science - Hyderabad Campus, Hyderabad, India; 2 Maternal and Child Health and Nutrition, ICMR–National Institute of Nutrition (NIN), Jamai Osmania PO, Hyderabad, 500007, India, +91 4027197241

**Keywords:** nutrition surveillance, institute-based monitoring, overweight, obesity, metabolic risk factors, malnutrition, life course, adolescents, first 1000 days, primary health care, double-duty actions, India, protocol, systems

## Abstract

**Background:**

India faces a complex spectrum of malnutrition, with undernutrition, micronutrient deficiencies, and rising overweight and obesity coexisting across populations and life stages. National surveys, such as the National Family Health Survey, Comprehensive National Nutrition Survey, and WHO STEPwise approach to Surveillance, provide essential population-level estimates but are episodic, limiting their utility for timely, institution-level prevention and early detection of nutrition and metabolic risks. Continuous, life course–oriented surveillance is required to support responsive public health action and double-duty approaches addressing multiple forms of malnutrition.

**Objective:**

This study aims to describe a life course framework and study protocol for an institute-based, standardized surveillance system to monitor overweight, obesity, and metabolic risk factors in India.

**Methods:**

This study protocol was developed through a structured synthesis of international surveillance models and public health frameworks, combined with operational insights from ongoing implementation initiatives in India. The proposed surveillance system is institution-anchored and spans key life stages from preconception to young adulthood. Core domains include anthropometry, diet, selected biochemical markers, and behavioral risk factors. Data collection is designed using a hybrid model incorporating optical mark recognition–based paper tools, digital platforms, and connected measurement devices, guided by principles of primary health care, including equity, community participation, intersectoral coordination, and appropriate technology.

**Results:**

The surveillance protocol has transitioned from conceptual development to implementation through 2 intramural research platforms: NULRISC (sanctioned July 2024) and COPRIME (sanctioned May 2025). Formative phase activities are ongoing in Telangana across approximately 15 high schools and colleges, as well as primary health care–linked platforms, including 3 primary health centers, 2 kindergartens, and 5 primary schools. At the time of manuscript revision, 10 educational institutions (5 schools and 5 colleges) were onboarded, with expansion to 30 institutions planned. The first surveillance round is scheduled for June 2026 in Hyderabad and Medchal–Malkajgiri districts, and initial analytical outputs are anticipated in 2027.

**Conclusions:**

An institute-based, standardized surveillance system has the potential to shift nutrition and metabolic risk monitoring in India from episodic assessment to continuous prevention. By embedding surveillance within routine institutional platforms and aligning with primary health care principles, the proposed protocol offers a feasible and sustainable approach to strengthening evidence-informed policy and practice across the life course.

## Introduction

Overweight, obesity, and other forms of malnutrition now coexist as part of a continuum of nutritional imbalance that increasingly shapes global health profiles across the life course. Worldwide, the prevalence of overweight and obesity has nearly tripled since 1975, with more than 1 billion individuals affected in 2022, including an estimated 159 million children and adolescents [1]. This growing burden contributes substantially to noncommunicable diseases (NCDs) and interacts with persistent undernutrition and micronutrient deficiencies, particularly in low- and middle-income countries.

India exemplifies this complex epidemiological reality through what has been described as a “triple burden of malnutrition,” characterized by the simultaneous presence of undernutrition, micronutrient deficiencies, and rising overweight and obesity within the same populations, communities, and even households [2,3]. This coexistence poses distinct public health challenges, as early-life undernutrition and later-life excess energy intake often intersect to amplify cardiometabolic risk, perpetuating intergenerational cycles of poor health and inequity.

The concept of nutrition transition, first articulated by Popkin [4], provides a useful framework for understanding these shifts. Rapid economic growth, urbanization, and globalization have altered dietary patterns and physical activity environments in India, leading to increased consumption of refined grains, added sugars, edible oils, and ultra-processed foods, alongside declining intake of fruits, vegetables, and pulses [5]. These changes have been accompanied by reduced physical activity and increased sedentary behavior, contributing to a rising prevalence of obesity and diet-related NCDs, such as hypertension, type 2 diabetes, cardiovascular disease, and certain cancers. At the same time, undernutrition continues to predispose vulnerable groups to infections, low birth weight, stunting, and impaired cognitive development [6].

Evidence from life course epidemiology underscores that nutritional and behavioral exposures during early and transitional stages of life have long-term consequences for metabolic health. The period spanning preconception, pregnancy, and the first 1000 days is particularly critical for metabolic programming and future disease risk [7]. Subsequent stages—early childhood, school age, adolescence, and young adulthood—represent additional windows during which dietary patterns, physical activity, sleep, and other behaviors consolidate. The surveillance of nutrition and metabolic risk factors across these stages is therefore essential for identifying early deviations in energy balance, body composition, and lifestyle before they become entrenched and translate into adult NCDs [8].

India currently relies on several large-scale nutrition and health surveys, including the National Family Health Survey (NFHS), the Comprehensive National Nutrition Survey (CNNS), ICMR-INDIAB, and WHO STEPS, to generate population-level estimates of nutritional status and risk factors. While these surveys provide invaluable national benchmarks, they are largely cross-sectional and episodic, typically conducted at intervals of 5 years or longer. As a result, they lack the frequency, institutional linkage, and life-stage continuity required for timely local action, program monitoring, or rapid course correction. Moreover, substantial delays between data collection and dissemination—often exceeding 2 to 3 years—further limit their utility for preventive decision-making at institutional or district levels [9,10]. In contrast, infectious disease surveillance systems, such as India’s Integrated Disease Surveillance Programme, demonstrate that continuous, institution-anchored data flows are operationally feasible and can support timely public health responses [11].

Addressing obesity and other nutrition-related NCDs in India, therefore, requires a complementary surveillance approach that moves beyond episodic surveys toward routine, institute-based monitoring across the life course. Institutions such as kindergartens, Anganwadi centers, schools, colleges, and primary health centers offer stable platforms with defined populations, governance structures, and recurring contact points that are well suited for periodic surveillance. Embedding standardized surveillance within these settings can leverage existing national programs, such as Rashtriya Bal Swasthya Karyakram (RBSK), Rashtriya Kishor Swasthya Karyakram (RKSK), POSHAN 2.0, PM POSHAN, and the Ayushman Bharat School Health Programme, while also enabling the participation of private educational institutions to improve representativeness and equity.

In this context, this study describes a protocol for a life course, institute-based standardized surveillance system for overweight, obesity, and metabolic risk factors in India. The proposed protocol emphasizes appropriate and context-sensitive technology, including optical mark recognition (OMR)–based paper tools for school settings and digital or Internet of Things (IoT)-enabled platforms where feasible, to ensure scalability, data quality, and ethical oversight. Importantly, the protocol is informed by practical operational experience from ongoing intramural implementation initiatives at ICMR-National Institute of Nutrition, which provide early insights into feasibility, indicator selection, and system integration.

Despite the availability of multiple data sources, India currently lacks a coherent and sustainable framework that systematically links institutional screening, surveillance, and preventive action across life stages. Establishing an institute-based surveillance ecosystem offers the opportunity to transform fragmented data into continuous, actionable intelligence. Such a system can support the early detection of risk, enable double-duty actions addressing multiple forms of malnutrition, and strengthen evidence-informed policy and practice across the life course.

## Methods

### Study Design and Purpose

This study presents a study protocol for a life course, institute-based standardized surveillance system to monitor overweight, obesity, and metabolic risk factors in India. The proposed system is designed as a continuous, periodic, and institution-anchored surveillance mechanism rather than a one-time survey or intervention trial. Its primary purpose is to generate timely, actionable data across key life stages to support early risk identification, preventive counseling, program monitoring, and policy feedback.

The protocol emphasizes routine surveillance embedded within existing institutional platforms, including kindergartens, Anganwadi centers, schools, colleges, primary health centers, and selected workplaces, to complement large-scale national surveys by providing higher-frequency, institute-level intelligence. The design is informed by principles of primary health care (PHC), including equity, community participation, intersectoral coordination, and appropriate use of technology.

### Development of the Conceptual Framework

The conceptual framework underpinning this surveillance protocol was developed through a structured synthesis of international surveillance models, relevant public health theories, and operational experience from ongoing implementation projects in India. Given the complex and coexisting forms of malnutrition observed across the Indian population, a multitheoretical and systems-oriented approach was adopted.

### Review of Existing Surveillance Models

A targeted review of established international surveillance and cohort systems was undertaken to identify design features relevant to a life course, institute-based approach. The models reviewed included the Youth Risk Behavior Surveillance System (YRBS), the National Health and Nutrition Examination Survey (NHANES), the Environmental Influences on Child Health Outcomes (ECHO) program, the WHO European Childhood Obesity Surveillance Initiative, and the Global School Health Policies and Practices Survey [12-16].

These models informed key elements of the proposed protocol, including periodic data collection; standardized indicators; integration of anthropometric, behavioral, and biological measures; and the use of institutional platforms as units of observation and action. However, none of these systems individually addressed the dual requirements of life course coverage and routine institute-level surveillance within the Indian context, necessitating contextual adaptation and integration.

### Theoretical Foundations

Five complementary theoretical perspectives were integrated to guide the design and operational logic of the surveillance system.

Nutrition transition theory provides a lens to understand the simultaneous rise in overweight and obesity alongside persistent undernutrition in India, driven by rapid dietary shifts, urbanization, and changing physical activity patterns [4,17]. Surveillance systems must therefore be capable of capturing both energy excess and nutrient deficiency within the same populations.

The life course perspective emphasizes that nutritional and behavioral exposures from preconception through young adulthood influence long-term metabolic risk. Surveillance across sequential life stages, including preconception, pregnancy, the first 1000 days, early childhood, school age, adolescence, and young adulthood, enables the early detection of risk trajectories and supports timely preventive action.

The social ecological model recognizes that health behaviors are shaped by interactions across individual, interpersonal, institutional, community, and policy levels [18]. Institute-based surveillance operates primarily at the institutional level, where schools, colleges, health facilities, and workplaces provide structured environments for measurement, feedback, and linkage to program action.

Behavior change communication models, including the health belief model and the social cognitive theory, highlight the role of awareness, perceived risk, and self-efficacy in behavior modification [19,20]. Surveillance systems that provide individualized and institution-level feedback, such as growth charts, dietary quality indicators, or physical activity summaries, can therefore function not only as monitoring tools but also as catalysts for behavioral change.

Finally, the surveillance-as-action paradigm reframes surveillance as an active public health function, in which data collection is explicitly linked to response, accountability, and program refinement [11]. Lessons from infectious disease surveillance systems, such as India’s Integrated Disease Surveillance Programme, demonstrate the feasibility of decentralized, real-time reporting mechanisms that inform timely action. Extending this paradigm to nutrition and NCD risk surveillance requires designing systems that are responsive, continuous, and locally actionable.

### Integrative Framework

**Figure 1.**
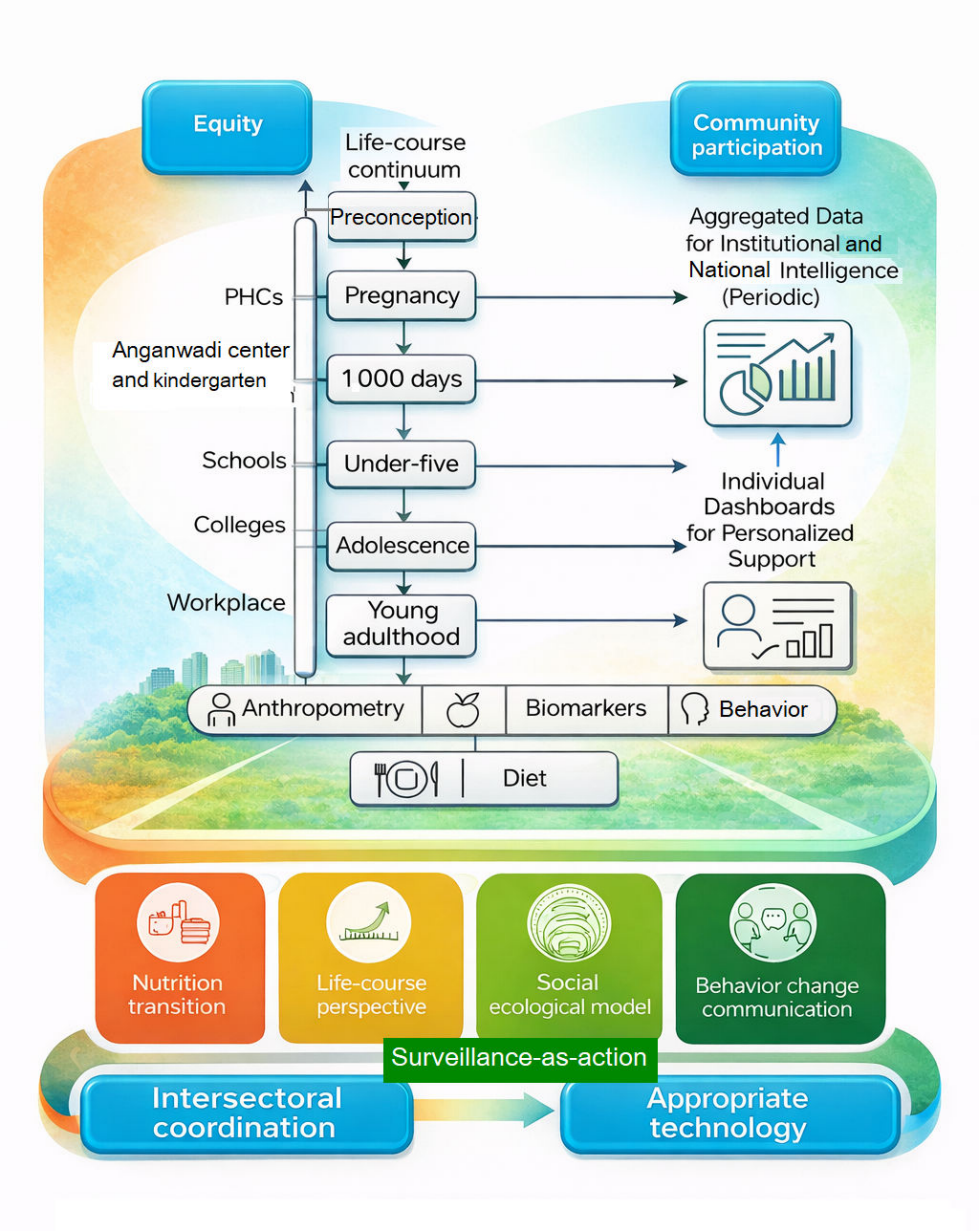
Conceptual framework for the institute-based standardized surveillance system of risk factors for overweight, obesity, and other forms of malnutrition in India. PHCs: primary health centers.

Integrating these theoretical perspectives yields a multidimensional framework for institute-based surveillance (Figure 1). The nutrition transition theory defines the evolving drivers of risk, the life course perspective identifies critical windows for monitoring, the social ecological model situates institutions as operational nodes, behavior change communication models ensure engagement through feedback, and the surveillance-as-action paradigm embeds responsiveness and accountability. Together, these elements provide a coherent foundation for designing a technology-enabled, life course surveillance system aligned with PHC principles.

The framework integrates 5 theoretical lenses (nutrition transition theory, life course perspective, social ecological model, behavior change communication models, and surveillance-as-action paradigm) within the 4 principles of PHC (equity, community participation, intersectoral coordination, and appropriate technology). Institutions, such as Anganwadi centers, schools, colleges, and primary health care facilities, serve as sentinel nodes for the periodic collection of anthropometric, dietary, biochemical, and behavioral indicators. The data are processed through hybrid OMR, digital, and IoT technologies, feeding upward into district and national dashboards, while feedback loops ensure local action and double-duty nutrition interventions across life stages.

### Operational Refinement and Contextualization

The protocol design was further refined through practical insights from initial implementation phases of intramural projects funded by the Indian Council of Medical Research. These experiences informed decisions related to indicator selection, technology mix, data flow architecture, quality assurance mechanisms, and ethical safeguards.

No new human participant data were collected for the preparation of this study. Ethical approvals for the referenced implementation projects were obtained in accordance with the Indian Council of Medical Research guidelines.

### Study Setting and Surveillance Units

India has generated substantial evidence on population nutrition and health status through large-scale surveys, such as the NNFHS, the CNNS, the National NCD Monitoring Survey, and WHO STEPS. These surveys have produced nationally representative estimates of anthropometry, dietary patterns, and behavioral risk factors and remain indispensable for benchmarking and policy planning [2,6,21,22]. However, their episodic nature—typically conducted at intervals of 5 years or longer—creates substantial temporal gaps, limiting their usefulness for continuous monitoring, institutional feedback, and timely preventive action.

In addition, existing program platforms, such as the RBSK and the RKSK, provide important opportunities for screening and counseling among children and adolescents. Nevertheless, the data generated through these platforms are largely clinical, fragmented across sectors, and seldom integrated into longitudinal nutrition or metabolic risk surveillance systems [23]. As a result, despite a strong foundation for population-level assessment, India currently lacks a routine, institute-anchored surveillance mechanism that connects educational and health institutions as recurring observation points within communities.

### Limitations of Survey-Based Monitoring

While national surveys, such as the NFHS and the CNNS, are invaluable for estimating prevalence and trends at state and national levels, they are not designed to support routine institutional feedback, early detection of emerging risk patterns, or rapid program adjustment. Large sampling frames and complex field operations constrain their applicability for local decision-making, and delays between data collection, analysis, and dissemination frequently exceed 2 to 3 years [9]. Furthermore, many surveys prioritize women of reproductive age and children under 5 years, resulting in the relative underrepresentation of adolescents (10‐19 y) and young adults (20‐24 y)—life stages that are critical for the consolidation of dietary, physical activity, and other behavioral risk factors influencing long-term NCD risk [24].

By contrast, infectious disease surveillance systems, such as India’s Integrated Disease Surveillance Programme, demonstrate that continuous, institution-based data flows are operationally feasible and can support early detection and timely response [11]. Applying similar surveillance logic to nutrition and metabolic risk factors offers an opportunity to address a major systems gap in NCD prevention.

### Rationale for an Institute-Based Surveillance Approach

Institutions—including kindergartens, Anganwadi centers, schools, colleges, primary health centers, and selected workplaces—represent stable environments with defined populations, established governance structures, and recurring service or academic calendars. Anchoring surveillance within these institutions offers several operational advantages.

First, *sustainability* is enhanced by aligning data collection with routine institutional cycles, reducing dependence on externally funded survey rounds. Second, *continuity* is facilitated through repeated measurements within the same institutions, enabling longitudinal assessment of trends and early identification of deviations in anthropometry, dietary quality, or metabolic risk markers. Third, *inclusivity* is strengthened by integrating both public and private institutions, improving representation across socioeconomic groups. Finally, *actionability* is increased, as institution-level data can directly inform local health promotion activities, school meal planning, counseling services, and community interventions.

In educational settings, surveillance may use OMR-based paper tools—similar in principle to systems, such as the Youth Risk Behavior Surveillance System—allowing standardized data collection in contexts with limited digital infrastructure [25]. In health care institutions and workplaces, digital or IoT-enabled platforms can be used where feasible to support real-time data capture and integration.

### Life Course Relevance of Institutional Platforms

An institute-based surveillance system is inherently life course oriented. During adolescence and young adulthood, schools and colleges provide access to large populations for monitoring body mass index, dietary patterns, physical activity, and other behavioral risk factors. In the preconception and pregnancy periods, primary health centers and Anganwadi centers serve as key surveillance nodes for indicators, such as maternal nutritional status, gestational weight gain, anemia, and gestational diabetes. For infants and young children, growth monitoring and infant and young child feeding indicators can be integrated within existing Integrated Child Development Services (ICDS) and POSHAN 2.0 platforms [26].

This institutional continuum enables the systematic documentation of nutritional exposures across successive life stages and facilitates linkage between early-life risk factors and later adolescent or adult outcomes. By embedding surveillance within institutions that individuals routinely access, the system supports repeated observation without imposing additional survey burden.

### Standardization and Interoperability

The proposed protocol emphasizes standardized anthropometric and behavioral indicators, uniform data formats, and interoperable digital dashboards across institutions. The data are envisioned to flow upward from classrooms and clinics to district and state repositories, creating a distributed yet integrated surveillance architecture. Periodic, nonstigmatizing feedback reports at institutional and district levels are intended to transform surveillance from a descriptive exercise into an actionable public health function, supporting double-duty actions that address both undernutrition and overweight.

### Target Population and Life Course Stratification

The surveillance protocol adopts a life course approach, structured around institutions that serve as fixed and recurring community anchors, including Anganwadi centers, schools, colleges, workplaces, and primary health care facilities (Table 1). These institutions function as sentinel surveillance units, periodically capturing nutrition-related and NCD-related risk indicators and generating data for both local action and higher-level aggregation. The system design aligns with World Health Organization recommendations for public health surveillance systems to be continuous, simple, flexible, acceptable, and action-oriented [16,22].

**Table 1.** Life course surveillance framework for overweight, obesity, and metabolic risk factors in India.

Life stage	Typical institution	Priority indicators	Surveillance purpose
Preconception	Community platforms, Eligible Couple Register, preconception counseling platforms	BMI, hemoglobin, dietary adequacy, physical activity, menstrual health	Early detection of undernutrition and overnutrition prior to conception
Pregnancy and lactation	PHC^a^, antenatal clinics	Gestational weight gain, anemia, GDM^b^, HDP^c^, dietary intake	Early identification of maternal risk and counseling
First 1000 days (0‐2 y)	PHC, Anganwadi centers	Breastfeeding, complementary feeding, infant growth	Strengthening IYCF^d^ practices and growth monitoring
Under-five (0‐5 y)	ICDS^e^, preschools (kindergarten)	Weight-for-height, MUAC^f^, anemia, dietary diversity	Monitoring wasting and stunting trajectories
School age (6‐9 y)	Primary schools	BMI-for-age, diet, physical activity, screen time	Identification of emerging overweight patterns
Adolescents (10‐19 y)	Schools, colleges	BMI-for-age, WHtR^g^, diet, physical activity, body image, substance use	Informing life-skills education and prevention
Young adults (20‐24 y)	Colleges, workplaces	BMI, waist circumference, blood pressure, stress, diet, sleep	Early detection of metabolic risk

a PHC: primary health center.

b GDM: gestational diabetes mellitus.

c HDP: hypertensive disorders of pregnancy.

d IYCF: infant and young child feeding.

e ICDS: Integrated Child Development Services.

f MUAC: mid-upper arm circumference.

g WHtR: waist-to-height ratio.

Surveillance windows are defined across the continuum from preconception to young adulthood, consistent with the first 1000 days framework and the adolescent-youth continuum [22,27]. Stratification by life stage enables the identification of critical periods for early risk detection, counseling, and preventive intervention.

This institution-anchored stratification supports the continuity of surveillance across life stages while leveraging existing human resources, such as teachers, auxiliary nurse midwives, and accredited social health activists, thereby minimizing additional operational burden.

### Indicators and Measurement Domains

To ensure comparability across institutions and over time, the protocol proposes a standardized core indicator set, supplemented by context-specific expanded indicators where resources permit. Indicators are organized into four primary domains.

#### Anthropometric Indicators

Core anthropometric measures include height, weight, BMI (or BMI-for-age *z* scores), waist circumference, and waist-to-height ratio. Mid-upper arm circumference and triceps skinfold thickness may be included as supplemental measures in selected settings [28].

#### Dietary Indicators

Dietary assessment includes simplified 24-hour dietary recall or food-frequency modules, diet diversity scores, and frequency of consumption of ultra-processed foods. In school and household contexts, basic food environment mapping may be conducted to contextualize dietary patterns [29].

#### Biochemical Indicators

Biochemical assessment focuses on hemoglobin as a core indicator, with periodic or point-of-care measurement of fasting glucose, lipid profile, hemoglobin A_1c_, ferritin, and other markers, such as urinary iodine or thyroid function tests, included where feasible and ethically appropriate [30].

#### Behavioral and Environmental Indicators

Behavioral indicators include physical activity (using adapted Global Physical Activity Questionnaire [GPAQ] or Youth Risk Behavior Surveillance System [YRBS] modules), sedentary behavior and screen time, sleep duration, tobacco and alcohol use, perceived stress, and body image perception [15,25].

Core indicators include height, weight, BMI (or BMI-for-age), waist circumference, hemoglobin, key dietary quality markers, and physical activity. Expanded indicators, such as biochemical markers, skinfold thickness, or detailed dietary modules, are included in selected institutions based on feasibility and ethical approval.

Together, these domains capture the balance between energy intake and expenditure, behavioral determinants, and biological manifestations that define malnutrition in all its forms.

### Data Capture Tools and Technology Architecture

The surveillance system uses a hybrid technology model designed to accommodate India’s heterogeneous digital infrastructure.

#### OMR-Based Paper Tools

In school and college settings, OMR-based paper tools enable standardized, low-cost data capture at scale, without reliance on continuous internet connectivity. Completed forms can be processed using locally available scanners, allowing rapid aggregation while preserving privacy, analogous to systems such as the YRBS [25].

#### Digital Platforms and Web Portals

In urban institutions and primary health care facilities, app-based or web-based platforms may be used to facilitate real-time data entry, automated dashboards, and the generation of individual or group-level feedback reports. Where feasible, interoperability with Ayushman Bharat Digital Health Mission identifiers may be explored to support longitudinal linkage.

#### IoT-Enabled Measurement Devices

Digital weighing scales, stadiometers, blood pressure monitors, glucometers, and selected point-of-care devices can be integrated to directly upload measurements to secure databases, reducing transcription errors and improving data quality [31].

#### Data Flow and Feedback Loops

The data collected at the institutional level are transmitted to district and state repositories through secure, encrypted app programming interfaces, with provisions for offline storage and periodic synchronization in low-connectivity settings. After validation and aggregation, automated, nonstigmatizing feedback reports are generated for institutions, PHC teams, and caregivers to enable local interpretation and timely action. These reports support targeted counseling, referral linkage, and planning of school-level and PHC-level health promotion activities, thereby operationalizing the surveillance-as-action paradigm within routine institutional workflows. Model one-page feedback report formats for educational institutions and for PHC-Anganwadi platforms are provided as MultimediaAppendices 1  2.

### Ethical Considerations

Ethical safeguards are integral to the acceptability and sustainability of the surveillance system. Key provisions include informed consent and assent procedures, particularly for minors; confidentiality through anonymization and restricted access to identifiable data; and delivery of feedback using nonstigmatizing language to minimize the risk of labeling or harm [32]. Data governance will be overseen by institutional ethics committees and aligned with the Digital Personal Data Protection Act, 2023 [33]. An explicit equity lens will guide implementation to ensure the inclusion of low-resource institutions and marginalized populations.

### Integration With Existing National Programs and Systems

#### Rationale for Program Integration

The proposed institute–based surveillance protocol is designed to complement and strengthen existing national nutrition and health programs rather than duplicate or replace them. By providing a standardized, institution-anchored surveillance layer, the protocol enables periodic data generated across programs to be consolidated into a coherent life course monitoring system. This integration is intended to reduce fragmentation, improve data use for prevention, and support double-duty actions addressing both undernutrition and overweight and obesity [34].

#### Rashtriya Bal Swasthya Karyakram

The RBSK provides a nationwide platform for screening children from birth to 18 years through mobile health teams and district early intervention centers. Within this protocol, RBSK platforms can serve as surveillance nodes for anthropometric and selected metabolic risk indicators among school-age children and adolescents. The standardization of nutrition-related measures, such as BMI-for-age, waist circumference, and selected dietary and physical activity indicators, would enable a shift from primarily curative screening toward preventive, trend-based surveillance while remaining aligned with the RBSK’s existing operational workflows [23].

#### Rashtriya Kishor Swasthya Karyakram

The RKSK targets adolescents aged 10 to 19 years through school-based and community-based platforms, including adolescent-friendly health clinics and peer educator networks. The integration of the surveillance protocol within the RKSK would enable periodic, gender-disaggregated monitoring of anthropometry, anemia, dietary patterns, physical activity, and other lifestyle risk factors relevant to adolescent health [35]. This linkage supports continuity between school-based surveillance and health-facility–based counseling, addressing a critical life stage during which behavioral and metabolic risk trajectories are established.

#### POSHAN 2.0 and the ICDS Platform

Mission POSHAN 2.0 consolidates ICDS, Anganwadi services, and the Poshan Tracker platform for growth and nutrition monitoring during pregnancy, early childhood, and the first 1000 days. The institute-based surveillance protocol is designed to interface with POSHAN 2.0 data streams by aligning indicator definitions, periodicity, and reporting formats. This interoperability enables under-five and maternal nutrition data, such as stunting, wasting, anemia, and dietary diversity, to be analytically linked with school-level and adolescent-level surveillance, facilitating life course continuity across the malnutrition spectrum [36].

#### PM POSHAN (School Meal Program)

The PM POSHAN Scheme provides daily meals to a large population of primary and upper-primary school children. Embedding surveillance within PM POSHAN–covered schools allows linkage between nutritional inputs (meals) and outcomes (anthropometry, diet quality, and physical activity indicators). Periodic feedback from school-based surveillance can inform menu planning, fortification strategies, and hygiene practices. The inclusion of private and unaided schools—currently outside PM POSHAN—within the surveillance protocol is proposed to enhance representativeness and enable comparative analysis across socioeconomic groups [36].

#### Ayushman Bharat and Digital Health Integration

Ayushman Bharat, through Health and Wellness Centres and the School Health Programme, provides a platform for preventive and promotive care across the life course. Within this protocol, Health and Wellness Centres function as data aggregation, validation, and counseling hubs, while school health teams support periodic surveillance rounds using standardized tools. Where feasible, linkage with Ayushman Bharat Digital Health Mission identifiers enables interoperability and longitudinal tracking, subject to ethical approvals and data governance safeguards [37].

#### Private-Sector and Institutional Partnerships

To improve inclusivity and sustainability, the protocol incorporates the voluntary participation of private schools, colleges, and workplaces, which collectively serve a substantial proportion of adolescents and young adults in India [38]. Partnerships with private hospitals, academic institutions, and laboratories are envisioned for capacity building, biomarker validation, and quality assurance. Such public-private collaboration strengthens surveillance coverage while maintaining standardized protocols and ethical oversight.

#### Enabling Double-Duty Actions and Policy Convergence

By integrating data across maternal, child, adolescent, and young adult platforms, the surveillance system enables double-duty actions that address multiple forms of malnutrition simultaneously. Consolidated dashboards support the identification of convergent risk patterns, such as early-life undernutrition followed by adolescent overweight, informing coordinated responses across food systems, physical activity promotion, and health services. This approach aligns with global and national commitments to double-duty actions and the Sustainable Development Goals (SDGs) related to nutrition and NCD prevention [34,39].

#### Summary

Integration with existing national programs positions the institute-based surveillance protocol as a unifying operational layer across health, nutrition, education, and welfare systems. By standardizing indicators, aligning data flows, and incorporating both public and private institutions, the protocol supports a scalable, inclusive, and life course–oriented surveillance architecture capable of generating actionable nutrition intelligence in real time.

### Alignment With Primary Health Care Principles

#### Overview

The institute-based surveillance protocol is explicitly grounded in the principles of primary health care as articulated in the Alma-Ata Declaration and reaffirmed in the Astana Declaration [40,41]. These principles—equity, community participation, intersectoral coordination, and appropriate technology—are operationalized within the design, implementation, and governance of the surveillance system to ensure that data generation and use remain people-centered, ethically grounded, and action-oriented.

#### Equity

Equity is a foundational consideration in the surveillance design, recognizing the pronounced social gradients in malnutrition and metabolic risk across populations. The protocol embeds surveillance within both public and private institutions, including Anganwadi centers, government schools, private schools, colleges, and workplaces, to ensure representation across socioeconomic strata. By standardizing indicators and data formats across institutions, the system enables the identification of high-risk clusters while supporting equitable resource allocation and program targeting.

To minimize exclusion related to digital access, the protocol uses context-appropriate technology. OMR-based paper tools are used in resource-constrained settings, while digital platforms and connected devices are used where infrastructure permits. This tiered approach is intended to prevent the widening of the digital divide and to position surveillance data as a public good accessible for population-level planning and prevention [10,29].

#### Community Participation

Consistent with PHC principles, the protocol treats communities not merely as sources of data but as active participants in surveillance and response. Implementation involves the engagement of students, parents, teachers, accredited social health activists, and Anganwadi workers in data collection, interpretation, and feedback processes.

In educational settings, age-appropriate self-report modules adapted from established surveillance instruments are used to capture dietary and lifestyle behaviors [25]. Automated, nonstigmatizing summary reports are generated for parents, institutions, and local health teams to support counseling and informed decision-making. The periodic dissemination of aggregated findings through community platforms, such as nutrition campaigns and health and wellness activities, closes the feedback loop between surveillance and behavior change. This participatory design supports transparency, trust, and sustained compliance.

#### Intersectoral Coordination

Malnutrition and metabolic risk are shaped by determinants spanning health, education, nutrition, and broader social systems. The surveillance protocol incorporates mechanisms for intersectoral coordination by aligning institutional roles across sectors. Primary health centers and Health and Wellness Centres support the validation of anthropometric and biochemical data, schools and colleges operationalize periodic data collection, nutrition programs provide contextual linkage to food and feeding environments, and digital health platforms facilitate secure data integration and reporting.

Such coordination enables surveillance findings to inform actions beyond the health sector, including school meal planning, physical activity promotion, and community-level nutrition interventions. By structuring data flows and responsibilities across sectors, the protocol operationalizes PHC’s emphasis on collaborative action on the social determinants of health [41].

#### Appropriate Technology

The protocol applies the principle of appropriate technology by combining scientific validity with social acceptability and economic feasibility [42]. A hybrid technology model is adopted to suit diverse institutional contexts. OMR-based paper tools enable low-cost, scalable data capture in settings with limited digital infrastructure. IoT-enabled measurement devices, such as digital weighing scales, blood pressure monitors, glucometers, and portable hemoglobin meters, support accurate data capture in health care facilities. Digital dashboards and mobile apps facilitate real-time visualization and decision support for district and institutional stakeholders.

This flexible approach ensures that technology enhances, rather than replaces, human judgment and institutional capacity, allowing the surveillance system to scale while remaining responsive to local constraints [31].

#### Summary

By embedding equity, community participation, intersectoral coordination, and appropriate technology into its operational design, the institute-based surveillance protocol translates PHC principles into measurable practice. This alignment ensures that surveillance data are ethically generated, locally relevant, and actionable, supporting prevention-oriented responses to malnutrition and metabolic risk across the life course.

### Operational Workflow for Institutional Surveillance

The surveillance workflow is designed as a station-based model aligned with the academic or service delivery schedule of the institution. On a typical school surveillance day, students are sequentially routed through 4 stations: registration and unique identifier assignment; anthropometric measurements; self-administered or interviewer-assisted behavioral and dietary assessment; and, where applicable, biochemical assessment.

Each station is managed by trained personnel, including teachers or institutional staff, project technical staff, and health functionaries. Anthropometric measurements are conducted using standardized protocols, and the average time per student is estimated at 5 to 7 minutes. A team of 4 to 6 trained personnel can complete measurements for approximately 120 to 150 students per day, depending on the institutional schedule and the availability of space.

Surveillance periodicity varies by life stage and institutional platform, with annual or biennial rounds in schools and colleges, and more frequent measurements integrated within routine PHC contacts for maternal and early childhood platforms.

### Data Flow and Data Governance

Data captured through OMR-based tools or digital devices undergo field-level validation, including completeness checks and basic plausibility screening. Digitized data are transmitted through secure, encrypted channels to a central server. In settings with limited internet connectivity, offline data storage with periodic batch synchronization is used.

Each participant is assigned a unique surveillance identifier to enable longitudinal linkage while maintaining confidentiality. Role-based access controls define permissions for data entry, validation, analysis, and dashboard visualization.

Automated dashboards generate institution-level summaries, which are shared with authorized stakeholders. Individual-level reports are accessible only to designated health personnel for counseling and referral.

Data retention follows institutional and national data governance policies, with deidentified datasets used for analysis and reporting.

### Quality Assurance and Standardization

All measurement devices are calibrated at predefined intervals. A minimum of 5% of anthropometric measurements are repeated for intraobserver and interobserver reliability assessment. Automated data checks flag biologically implausible values and missing fields at the point of digitization.

Quality indicators for each surveillance round include coverage, completeness of core variables, the proportion of implausible values, and turnaround time from data collection to feedback generation.

### Phased Implementation and Pilot Evaluation

The surveillance system will be implemented in a phased manner, beginning with sentinel institutions in Hyderabad and the Medchal–Malkajgiri districts of Telangana. A pilot evaluation will assess feasibility and system performance using predefined indicators, including institutional coverage, proportion of complete records, turnaround time for feedback reports, stakeholder acceptability, and the proportion of identified high-risk individuals linked to counseling or referral services.

The findings from the pilot phase will be used to refine workflows, training modules, and technology architecture prior to scale-up.

## Results

The institute-based surveillance protocol has transitioned from conceptual development to implementation through 2 intramural research platforms at the ICMR-National Institute of Nutrition.

The NULRISC project (sanctioned in July 2024) represents the school-based and college-based implementation arm of the surveillance framework. Formative phase activities, including stakeholder engagement, tool refinement, workflow standardization, and feasibility assessments, are currently underway in approximately 15 high schools and colleges in Telangana.

The COPRIME project (sanctioned in May 2025) operationalizes the early life course and primary care components of the surveillance model. Formative implementation is ongoing in 3 primary health centers, 2 kindergartens, and 5 primary schools to standardize measurement protocols, data flow architecture, and referral linkages.

At the time of study revision, 5 schools and 5 colleges have been formally onboarded as sentinel surveillance institutions, with a target of 30 institutions in the first implementation phase across the Hyderabad and Medchal–Malkajgiri districts of Telangana.

The first periodic surveillance round is scheduled for June 2026. The initial phase will generate institution-level coverage estimates, data completeness indicators, turnaround time metrics, and feasibility and acceptability assessments. Analytical outputs from the first surveillance cycle are expected in 2027.

## Discussion

### Principal Findings

The increasing prevalence of overweight, obesity, and other forms of malnutrition among Indian children, adolescents, and young adults underscores the need for surveillance systems that are continuous, standardized, and embedded within routine institutional settings. While national surveys remain essential for population-level benchmarking, their episodic design limits their ability to support early detection, local decision-making, and preventive action. The institute-based surveillance protocol presented in this paper addresses this gap by proposing a life course–oriented, institution-anchored system that links data generation directly to action across education, health, and nutrition platforms.

### Value of an Institute-Based Surveillance Model

Anchoring surveillance within institutions, such as Anganwadi centers, schools, colleges, primary health centers, and workplaces, offers several advantages over traditional survey-based approaches. First, repeated measurements aligned with institutional calendars enable timely monitoring of trends, allowing emerging risks to be identified before they consolidate into established disease patterns. Second, the protocol spans critical life stages, from preconception and the first 1000 days through adolescence and young adulthood, supporting a life course perspective that is essential for interrupting intergenerational cycles of malnutrition and metabolic risk [24,27].

Third, institution-level data enhance local relevance and usability, enabling context-specific responses such as school meal modifications, targeted nutrition counseling, or physical activity promotion. Fourth, integration across platforms, such as RBSK, RKSK, POSHAN 2.0, and PM POSHAN, enables double-duty actions that simultaneously address undernutrition and overweight, aligning with global recommendations for integrated malnutrition prevention [34]. Finally, the hybrid use of OMR-based paper tools and digital or IoT-enabled technologies supports cost-efficient scalability while remaining sensitive to infrastructural diversity and ethical considerations [31].

### Positioning Within Global Surveillance Practice

International experience demonstrates the feasibility and utility of institution-based and school-linked surveillance systems for child and adolescent health. Systems, such as the Youth Risk Behavior Surveillance System in the United States and the WHO European Childhood Obesity Surveillance Initiative, illustrate how standardized, low-burden data collection in schools can generate actionable insights at scale [25,43]. The NHANES and the ECHO further demonstrate the value of integrating behavioral, anthropometric, and biological measures within continuous or longitudinal frameworks [13,14].

Compared with these systems, the proposed institute-based model is distinguished by its explicit life course coverage, its integration of both public and private institutions, and its alignment with existing national programs rather than stand-alone surveillance infrastructure (Table 1). By combining behavioral, anthropometric, and selected biochemical indicators within a unified framework, the protocol adapts global best practices to India’s epidemiological and institutional context while retaining international comparability.

### Anticipated Implementation Challenges

Despite its conceptual and operational strengths, the implementation of the proposed surveillance system may encounter several challenges. Capacity constraints related to the training and supervision of teachers, frontline health workers, and institutional staff may affect the consistency of data collection. Ensuring data quality and standardization across diverse settings will require robust calibration protocols, periodic validation, and automated data checks. Ethical concerns, including the protection of minors’ data, informed consent, and the prevention of stigma, necessitate strong governance mechanisms and careful design of feedback processes [32].

Interoperability across existing program databases and the inclusion of private institutions will require sustained intersectoral coordination and alignment of data standards. Long-term sustainability will depend on policy commitment and financing mechanisms that transition surveillance from time-limited projects to routine institutional practice. These challenges are not unique to this protocol and can be mitigated through phased implementation, pilot testing within sentinel sites, and integration with digital public infrastructure and existing program workflows.

### Implications for Policy and Practice

By shifting nutrition and metabolic risk monitoring from episodic surveys to continuous, institution-anchored surveillance, the proposed protocol offers a pathway to strengthen preventive public health practice in India. The system is designed to generate timely, disaggregated evidence that can inform program refinement, resource allocation, and local action while maintaining alignment with national and global nutrition and NCD goals. If implemented at scale, this approach has the potential to transform how malnutrition in all its forms is monitored and addressed across the life course. The use of structured institutional feedback reports ensures that surveillance outputs are directly translated into decentralized preventive action within schools, Anganwadi centers, and PHC systems.

### Conclusion

India is at a critical juncture in its nutrition transition, with undernutrition, overweight, obesity, and diet-related NCDs coexisting within the same populations and life stages. Although national surveys continue to provide essential population-level estimates, their episodic design limits their capacity to support timely, local, and life course–specific preventive action. The institute-based standardized surveillance protocol presented in this paper offers a structured approach to addressing this gap.

By embedding surveillance within routine institutional settings—Anganwadi centers, schools, colleges, primary health centers, and workplaces—and aligning it with existing national programs, the proposed system reorients nutrition and metabolic risk monitoring from episodic assessment to continuous prevention. The use of a hybrid technology model combining OMR-based paper tools, digital platforms, and IoT-enabled devices enables scalability while remaining sensitive to infrastructural diversity and ethical considerations. Importantly, the protocol operationalizes the core principles of PHC—equity, community participation, intersectoral coordination, and appropriate technology—ensuring that surveillance functions as a public good rather than a purely technical exercise.

International experience from systems, such as YRBS, NHANES, ECHO, Global School Health Policies and Practices Survey, and the WHO European Childhood Obesity Surveillance Initiative, demonstrates that institutionalized surveillance is both feasible and impactful when integrated into education and health systems. Adapted to India’s context, the proposed life course surveillance model has the potential to generate timely, disaggregated evidence that informs double-duty actions, strengthens program convergence, and supports national commitments to the SDGs, particularly SDG 2 (Zero Hunger) and SDG 3 (Good Health and Well-being).

Reframing surveillance as a participatory, institute-anchored process transforms data collection into a continuous cycle of learning, accountability, and prevention. If implemented and sustained, this approach can contribute meaningfully to strengthening India’s public health response to malnutrition in all its forms across the life course.

## Supplementary material

10.2196/86084 Multimedia Appendix 1 Model institutional feedback report—institute-based nutrition and metabolic risk surveillance.

10.2196/86084 Multimedia Appendix 2 Model institutional feedback report—primary health center or Anganwadi nutrition and early life surveillance.

## Abbreviations

CNNS: Comprehensive National Nutrition Survey
ECHO: Environmental Influences on Child Health Outcomes
GPAQ: Global Physical Activity Questionnaire
ICDS: Integrated Child Development Services
IoT: Internet of Things
NCD: noncommunicable disease
NFHS: National Family Health Survey
NHANES: National Health and Nutrition Examination Survey
OMR: optical mark recognition
PHC: primary health care
RBSK: Rashtriya Bal Swasthya Karyakram
RKSK: Rashtriya Kishor Swasthya Karyakram
SDG: Sustainable Development Goal
WHO: World Health Organization
yrbs: YRBS is appearing twice
YRBS: Youth Risk Behavior Surveillance System
YRBS: Youth Risk Behavior Surveillance System
