# Supplementary material for: Life-Course Framework for Institute-Based Surveillance of Overweight, Obesity, and Metabolic Risk Factors in India: Protocol for Surveillance System Development
Source: JMIR Res Protoc. 2026 Apr 13;15:e86084. doi: 10.2196/86084 (PMC13075776; doi:10.2196/86084)
Supplement: Multimedia Appendix 1 [file resprot-v15-e86084-s001.pdf]

MODEL INSTITUTIONAL FEEDBACK REPORT

Institute-Based Nutrition & Metabolic Risk Surveillance

Confidential – For Institutional Use Only

Institution Name: Government High School, Nedchal

District: Medchal–Malkajgiri, Telangana

Date of Assessment: 18 June 2026

Class/Group Covered: Grades 8–10

Students Assessed: 412

Coverage: 94%

1. PURPOSE OF THIS REPORT

This report provides a summary of the nutrition and health indicators of students assessed in your institution. The findings are intended to support **health promotion, counselling, and school-level action**.
This is **not a diagnostic or ranking tool**.

2. KEY HIGHLIGHTS

- Most students are within the healthy growth range
- A small proportion require early lifestyle guidance
- Anaemia risk indicators observed in some students
- Physical activity levels below recommended levels in many students

3. GROWTH AND NUTRITION STATUS

BMI-for-age Classification

Category	% of Students
Normal range	68%
Thinness	11%
Overweight	14%
Obesity	7%

Interpretation: The majority of students are in the healthy range. A small proportion may benefit from nutrition and physical activity support.

4. KEY HEALTH AND LIFESTYLE INDICATORS

Dietary Pattern

- Daily fruit consumption: 32%
- Daily vegetable intake: 41%
- Ultra-processed food ≥3 times/week: 46%

Physical Activity

- ≥60 minutes/day: 38%
- <60 minutes/day: 62%

Screen Time (>2 hours/day)

- 57%

The names of villages, institutions, and facilities used in these model reports are fictitious and provided only for illustration. They do not refer to any actual location or service unit.

Haemoglobin (where assessed)

- Within normal range: 71%
- Suggestive of anaemia: 29%

---

5. WHAT THIS MEANS FOR YOUR INSTITUTION

These findings suggest opportunities to:

- Strengthen school physical activity periods
- Promote iron-rich and diverse foods
- Reinforce healthy snacking messages
- Integrate nutrition into school health activities

---

6. RECOMMENDED SCHOOL-LEVEL ACTIONS (NEXT 3–6 MONTHS)

✓ Ensure daily structured physical activity period

✓ Promote weekly “fruit & vegetable day”

✓ Nutrition and anaemia awareness session for students

✓ Engage School Health & Wellness Ambassador for lifestyle education

✓ Growth monitoring follow-up for students needing attention

---

7. STUDENT SUPPORT PATHWAY

Students identified for additional support:

- Received individual confidential health cards
- Referred to:
  - School Health Programme team / PHC
  - Counselling sessions (where required)

---

8. DATA QUALITY INDICATORS

Student participation: 94%

Form completeness: 97%

Measurement quality: Within acceptable limits

This indicates excellent institutional support.

---

9. NEXT SURVEILLANCE ROUND

Proposed timeline: June 2027

This will help track trends and assess improvements.

---

10. TECHNICAL SUPPORT

For planning health promotion activities, your institution may contact:

District Surveillance Unit / School Health Team

IMPORTANT NOTE

Individual student data are confidential and are **not included in this report**.

Only aggregated information is shared for institutional action.

The names of villages, institutions, and facilities used in these model reports are fictitious and provided only for illustration. They do not refer to any actual location or service unit.
