# Supplementary material for: Life-Course Framework for Institute-Based Surveillance of Overweight, Obesity, and Metabolic Risk Factors in India: Protocol for Surveillance System Development
Source: JMIR Res Protoc. 2026 Apr 13;15:e86084. doi: 10.2196/86084 (PMC13075776; doi:10.2196/86084)
Supplement: Multimedia Appendix 2 [file resprot-v15-e86084-s002.pdf]

MODEL INSTITUTIONAL FEEDBACK REPORT

PRIMARY HEALTH CENTRE / ANGANWADI NUTRITION & EARLY LIFE SURVEILLANCE

CONFIDENTIAL – FOR PROGRAMME STRENGTHENING

FACILITY NAME: PRIMARY HEALTH CENTRE, SHAJIRPET

LINKED ANGANWADI CENTRES: 12

DISTRICT: MEDCHAL–MALKAJGIRI, TELANGANA

ASSESSMENT PERIOD: MAY–JUNE 2026

1. POPULATION COVERED

GROUP	NUMBER ASSESSED	COVERAGE
PRECONCEPTION	132	96%
PREGNANT WOMEN	128	91%
LACTATING MOTHERS	96	88%
CHILDREN 0–2 YEARS	142	93%
CHILDREN 2–5 YEARS	176	89%

Coverage reflects strong frontline worker engagement.

(PRECONCEPTION: Adolescent girls / women of reproductive age)

2. PURPOSE OF THIS REPORT This report supports:

- Early identification of nutrition and metabolic risk across preconception, maternal, and early childhood stages
- Strengthening routine counselling
- Improving service delivery through POSHAN 2.0 and PHC platforms This is **not for individual diagnosis or performance ranking**.

3. MATERNAL AND PRECONCEPTION NUTRITION INDICATORS

Preconception Nutritional Status (Adolescent girls / women of reproductive age)

Indicator	Key finding
Underweight	16%
Normal BMI	68%
Overweight/obesity	16%

Anaemia status Within normal range: 58% Mild/moderate anaemia: 42%

Dietary adequacy Adequate dietary diversity: 44% Needs improvement: 56%

Interpretation: Opportunity to strengthen preconception screening, nutrition counselling, anaemia reduction, and healthy weight promotion before pregnancy.

Maternal Nutritional Status (Pregnant women)

Indicator	Key finding
Underweight	18%
Normal BMI	62%
Overweight/obesity	20%

Anaemia status: Within normal range: 54% Mild/moderate anaemia: 46%

Gestational weight gain (where applicable) Adequate: 48% Needs monitoring: 52%

Interpretation: Opportunity to strengthen early pregnancy nutrition counselling, anaemia management, and IFA adherence.

The names of villages, institutions, and facilities used in these model reports are fictitious and provided only for illustration. They do not refer to any actual location or service unit.

4. **INFANT AND YOUNG CHILD FEEDING (0–2 YEARS)**
    a. Early initiation of breastfeeding: 72%
    b. Exclusive breastfeeding (0–6 months): 64%
    c. Minimum dietary diversity (6–23 months): 38%

This indicates a need for focused complementary feeding counselling.

5. **CHILD GROWTH STATUS**

Children Under 5 Years

INDICATOR	% OF CHILDREN
**NORMAL GROWTH**	71%
**STUNTING RISK**	16%
**WASTING RISK**	9%
**OVERWEIGHT RISK**	4%

Most children are growing well; a small proportion require additional follow-up.

6. SERVICE DELIVERY OPPORTUNITIES

Based on the findings, the following actions may be prioritised:

✓ Early pregnancy BMI recording in all ANC registrations

✓ Intensified anaemia counselling and IFA follow-up

✓ Monthly complementary feeding demonstrations

✓ Targeted home visits for growth-falters

✓ Convergence with Village Health Sanitation & Nutrition Day

7. **REFERRAL AND FOLLOW-UP PATHWAY**

Individuals requiring additional care have been:
    a. Referred to Medical Officer / Staff Nurse at PHC
    b. Linked to nutrition counselling sessions
    c. Scheduled for follow-up growth monitoring All referrals are confidential.

8. **DATA QUALITY INDICATORS**

Record completeness: 95%

Anthropometry standardization: Within acceptable range

Timely data entry: 92%

This indicates good system readiness for routine surveillance.

9. **PROGRAMME CONVERGENCE USE**

This report can support:

- POSHAN 2.0 micro-planning
- Anaemia Mukt Bharat targeting
- High-risk pregnancy tracking
- VHSND counselling themes

10. NEXT SURVEILLANCE CYCLE

Proposed timeline: June 2027

Trend comparison will help assess programme impact.

TECHNICAL SUPPORT For action planning, please contact: Block PHC Surveillance Unit / ICDS Supervisor

The names of villages, institutions, and facilities used in these model reports are fictitious and provided only for illustration. They do not refer to any actual location or service unit.
